# College and Hospital Notes

**Published:** 1907-03

**Authors:** 


### COLLEGE AND HOSPITAL NOTES.

The Erie County Tuberculosis Hospital has been subjected to severe criticism of late. Dr. John H. Pryor, of Buffalo, an expert in the hospital treatment of consumptives, who established and superintended for some time the New York state hospital for the treatment of incipient tuberculous patients at Raybrook in the Adirondacks, lately presented a searching critique to the charity organisation society, which was referred to an investigating committee. The committee's report lately sent to the board of supervisors, sustains all the charges made by Dr. Pryor and comments exhaustively on the mismanagement of the hospital resultant from the ill considered action and want of action by the county legislators. The report is signed by W. Harry Glenny, chairman; Daniel J. Kenefick, the Rev. Samuel V. V. Holmes, Mrs. Melvin P. Porter, DeLancey Rochester, P. W. Van Peyma, Mrs. C. Peter Clark, Jessie H. Jewett, Julius Ullman, Simon Fleischmann, Mrs. Lucien Howe and Irving P. Lyon.

---

The University of Buffalo, all the departments participating, celebrated University Day February 22, 1907, with exercises at the Teck Theater appropriate to the occasion. A dinner of the associated alumni was given the previous evening at the Ellicott club. On the twenty-second the exercises began at 11 a. m. with an invocation by Bishop Walker, the vice-chancellor, Mr. Charles P. Norton, having previously called the vast assemblage to order. After instrumental and vocal music by the band and the university glee club Mr. Norton introduced Mr. J. N. Larned, who spoke at some length and with fervid eloquence, the text of his address being an argument for the establishment of a college of arts. The audience was a representative one composed of distinguished citizens, city officials, prominent women, alumni and undergraduates. University day was originally established by the alumni of the medical department February 22, 1899.

---

The New York Skin and Cancer Hospital announces that Dr. L. Duncan Bulkley will close his clinical course with four special lectures as follows: March 27, Practical Points in the Diagnosis and Treatment of Diseases of the Skin; April 3, Errors in Diagnosis and Treatment; Don'ts in Dermatology; April 10, Danger Signals from the Skin; April 17, The Significance and Treatment of Itching. Also a lecture by Dr. William Seaman Bainbridge, April 24, Some Phases of the Cancer Problem. Illustrated by a series of cases. In the Out-Patient Hall of the hospital, at 4:15 o'clock. The lectures will be free to the medical profession.

---

The Erie County Maternity Hospital was formally opened March 5, 1907. It is a stone building two and a half stories in height, containing a large circular ward, a sun room, a diet kitchen, a confinement or operating room and every modern improvement. The hospital staff consists of Dr. Regina Flood

Keyes, obstetrician; Dr. Carroll Roberts and Dr. Gurdon Potter. Miss Nellie Davis is the new superintendent and Miss Bertha Jones the assistant superintendent; Miss Dark is the assistant night superintendent.

---

The University of Buffalo has selected the last week in May in which to hold commencement ceremonies. The executive committee of the medical department alumni association has issued a preliminary circular inviting suggestions from the members as to the form and method of conducting the annual alumni meeting this year. It is important that early replies to this circular be made to the chairman of the Committee, Dr. Albert T. Lytle, 200 Lexington Avenue.

